# Gendered male and high-income country authors dominate publication at a One Health research organization

**DOI:** 10.1371/journal.pone.0352401

**Published:** 2026-06-26

**Authors:** Cecilia A. Sánchez, Collin J. Schwantes, Shannon Ball, Siyeun Kim, Sarah Munro, Rebecca Leaman, Cadhla Firth

**Affiliations:** EcoHealth Alliance, New York, United States of America; MIT, UNITED STATES OF AMERICA

## Abstract

Authorship on academic publications is consequential for researchers in science fields. One’s position in a list of authors is typically used to signal information about author contributions and status, with the first and last author positions regarded as the most prestigious and important for career advancement. Therefore, any inequities that exist in the allocation of authorship (e.g., associated with gender or geography) could affect researchers’ career progression. We assessed patterns in authorship at EcoHealth Alliance, a non-profit organization that conducted One Health and conservation research. We compiled a corpus of 451 peer-reviewed journal articles published from 2011–2022, each of which had at least one EcoHealth Alliance-affiliated author, and gathered information on the gender and country affiliation of authors in first and last author positions. Within the corpus, we found that gendered male researchers and researchers with high-income country (HIC) affiliations were often in prestigious author positions. Specifically, we found that gendered male authors represented 60% of first and last authors, 65% of first and last authorships (FLAs), and 91% of highly productive authors (those with ≥ 10 FLAs). Last authorships were particularly male-dominated, with 2.7 times as many last authorships by gendered male authors as by gendered female authors. Our network analysis revealed that gendered male authors were more structurally important to the author network on average and comprised 65% of highly “powerful” authors in the network. HICs were also overrepresented in the corpus, with 72% of FLAs listing an HIC affiliation. Though our analysis was based on articles with at least one EcoHealth Alliance-affiliated author, authorship affiliations in the corpus extended to nearly 250 institutions across 43 countries, suggesting broader applicability of our findings. We conclude by offering recommendations—informed by the patterns observed in our data and based on our personal experiences as researchers—that we believe would help address the gender and geography disparities in authorship patterns we observed.

## Introduction

Authorship on academic publications is consequential for researchers in science, technology, engineering, and mathematics (STEM), as it can bestow prestige, bolster reputations, and influence career trajectories [1]. An individual’s publication record is a key consideration in hiring, promotion, and tenure decisions [2–4]. Greater productivity in the form of authorship has also been linked to obtaining more research funding [5,6] and higher pay for STEM researchers [7]. However, the benefits of publication are not experienced equitably by those who contribute to a manuscript. In the sciences, author position (i.e., one’s position in a list of authors on a publication) often signals information about author contributions and status, with the first and last author positions typically (though not universally [8]) regarded as the most prestigious [9,10] and thus particularly important for career advancement [4,11]. Therefore, any inequities that exist in the allocation of authorship separate from actual contribution (e.g., associated with gender or geography) could affect researchers’ career progression [12,13].

Though differences exist between STEM fields, women are generally under-represented as authors, in overall number of authorships, and in first and last authorships [14–16]. For instance, a global study of scientific output found women accounted for < 30% of authorships and for every article with a female first author, there were nearly two by a male first author [14]. Gendered authorship gaps have slowly narrowed but disparities persist [15–17]. There is a widening gender gap in the last author position [15], which was likely exacerbated by the COVID-19 pandemic [18]. Though it is difficult to determine the mechanisms underlying gendered authorship disparities, suggested explanations include the slower career progression and/or attrition of women in science, an influence of gender in authorship negotiations, and differences in attribution and recognition of women’s contributions [15,17,19,20]. Notably, authors from low- and middle-income countries (LMICs) are also less likely to be listed as first or last authors on publications compared to authors from high-income countries (HICs) [21–23]. Even when research takes place in an LMIC or involves LMIC participants, researchers affiliated with that country are only included as first or last authors about half the time [24–26]. As a result, researchers with multiple marginalized identities are especially underrepresented as authors and in prestigious authorships. In a study of all publications by The Nature Conservancy (TNC), one of the world’s largest conservation non-profit organizations, women in the Global South represented 3% of all authors while first and last authorships by women in the Global South each comprised < 1% of all authorships [16].

Although there have been some efforts from publishers to promote more equitable and transparent recognition of scientific contributions in authorship (e.g., by requiring use of the Contributor Role Taxonomy (CRediT; [27]), it is also important for research organizations (particularly those in HICs) to critically examine their own authorship patterns and practices. Further, by making their findings public, organizations can spur broader study and reflection and contribute to an improved understanding of authorship norms in different scientific fields. Case studies of organizations that are highly influential in their fields are particularly valuable, as their practices may influence behavior of other organizations. The TNC case study above provides one such example and motivated the work we describe below.

All authors of this manuscript were formerly employed by EcoHealth Alliance (EHA), a non-profit research organization focused on pandemic prevention and wildlife conservation. Founded in 1971 as Wildlife Preservation Trust International (and later shortened to Wildlife Trust), the organization rebranded as EcoHealth Alliance in 2010 to reflect a transition from a conservation focus to a broader “One Health” agenda [28]. One Health is a concept that emphasizes the interconnectedness of animal, human, plant, and environmental health [29], and as a research field, it comprises diverse disciplines (e.g., ecology, biology, infectious disease, veterinary science, environmental science, public health, health policy). Although EHA was headquartered in New York City, most research projects were centered in LMICs (30 + countries). These projects were conducted in partnership with local researchers and guided by organizational principles such as collaboration, equity, diversity, and inclusion.

At the time this project was initiated (mid-2023), EHA employed ~50 scientific staff and ~10 administrative staff. Despite its modest size, EHA occupied an important role in the One Health and conservation research landscape. Projects at the organization were supported by federal funding (e.g., National Institutes of Health, National Science Foundation, Department of Defense, United States Agency for International Development) as well as grants from the private sector and foundations (e.g., Gates Foundation, Wellcome Trust, Samuel Freeman Charitable Trust). EHA scientists contributed to major scientific discoveries, including identifying viral reservoirs in wildlife and elucidating drivers of disease spillover and emergence, that were published in top-ranked journals such as Nature [30–32], Science [33], The Lancet ([34]), PNAS [35,36], The New England Journal of Medicine [37], and Emerging Infectious Diseases [38]. EHA also helped shape policy in public health and conservation through the involvement of its scientists with high-level panels and committees convened by organizations including the Intergovernmental Science-Policy Platform on Biodiversity and Ecosystem Services, the International Union for Conservation of Nature, the National Academies of Sciences, Engineering, and Medicine, and the World Organization for Animal Health.

Given EHA’s role as an HIC-based organization that partnered primarily with LMIC-based organizations, and an internal structure with majority women in early and middle career positions but majority men in leadership positions (authors’ personal observations), we felt that EHA provided an excellent opportunity to examine associations between gender, geography, and authorship. As such, we initiated a project to assess authorship patterns within EHA and its broader collaboration network. We hope this project will also motivate other internationally collaborating organizations to review their own authorship patterns. Importantly, we provide the de-identified data and code workflow underlying our analyses to serve as an initial template for such explorations.

## Materials and methods

### Ethical and organizational approval

On June 17, 2023, this study was approved as exempt by the Health Media Lab Institutional Review Board (Protocol #2264). This study was also approved by EHA senior leadership. Data were accessed for research purposes from June 18, 2023 through June 4, 2024. Authors had access to information that could identify individual participants during and after data collection.

### Data collation and cleaning

We compiled a set of peer-reviewed, EHA-affiliated journal articles published from January 1, 2011 to December 31, 2022 (henceforth, the *corpus*); see S1 Text for details. From this corpus, we developed three related tables of articles, authors, and authorships. Here, we use the term *author* to refer to a unique individual, and *authorship* to refer to an individual’s contribution to a specific journal *article*. By contributing to multiple articles, a single author therefore accumulates authorships over their career. We cleaned the authors table by removing duplicates, referring to sources such as ORCID (Open Researcher and Contributor ID), ResearchGate, and Google Scholar profiles to ensure that unique authors were identified as such despite minor differences in name spelling, use of initials, or accents. We also collected information on author gender (see *Gender classification of authors* below). We classified authorships as either first (including sole authorships), middle, or last, and restricted the authorships table to first and last authorships in recognition of the greater contribution and prestige associated with these positions [10]. In the case of co-first authorships, we included only the author listed first. All analyses described in this manuscript focus only on first and last authorships (FLAs) and do not include middle authorships.

We noted the country listed for each authorship affiliation (hereafter, *authorship geography*). If a single authorship listed multiple affiliations, we selected only the first, assuming it represented an organization or location of primary importance to the author. We classified each country as low-income, lower-middle-income, upper-middle-income, or high-income using historical World Bank data (https://datacatalogfiles.worldbank.org/ddh-published/0037712/DR0090754/OGHIST.xlsx). Because the World Bank classifies incomes annually, we used the most common designation for a country over the period of our dataset. For example, Bangladesh was classified by the World Bank as low-income from 2011–2013 and lower-middle-income from 2014–2022; therefore, we classified it as lower-middle-income. Finally, we assessed whether each article had a geographic focus; that is, the research described in the article required the authors’ physical presence in a country (e.g., for fieldwork) or specialized knowledge about a country. If yes, we recorded all focal countries of an article as the *article geography*. If the work described in an article could be performed regardless of location (e.g., a review or model-based article), we classified the article geography as “non-specific”.

### Gender classification of authors

We employed a two-pronged approach to gather author gender information (for authors with at least one first or last authorship; *n* = 498 authors).

1) **Pronouns-based approach.** Self-identified gender data are typically not publicly available. However, there is a growing practice of sharing one’s pronouns (e.g., she/her, he/him, they/them, she/they, they/he) in conversation and online (e.g., professional websites, email signatures) [39,40]. We therefore annotated author data with manually-gathered pronouns [41] to infer gender. To gather author pronouns, we first drew from professional interactions we have had with authors in the corpus (e.g., scientific collaborations, conference attendance, working groups). For authors not known to us, we searched online for publicly available information (e.g., lab websites, interviews, press releases, conference programs) that contained pronouns. Pronouns were recorded as “she/her/hers”, “he/him/his”, “they/them/their” (with the option to select multiple of these) or “unable to find”. We classified authors as *gendered female* if we only found evidence that they used she/her/hers pronouns, as *gendered male* if we only found evidence that they used he/him/his pronouns, and as *gendered nonbinary* if we found evidence they used they/them/their pronouns or any combination of he/she/they pronouns [42]. Here, we use *nonbinary* as “an umbrella term for people whose gender identity doesn’t sit comfortably with ‘man’ or ‘woman’” [43]. We chose *nonbinary* because the Gender Census 2024 (which surveyed nearly 50,000 participants) found it to be the term that is most preferred (60.4%) by individuals who do not identify as fitting into the male/female binary (https://www.gendercensus.com/results/2024-worldwide). We use the terms *gendered male* and *gendered female* to emphasize that these are externally imposed classifications [44], and acknowledge that a person’s gender does not necessarily correspond to the pronouns they use. However, using pronouns as a proxy for gender identity allowed us to include those with nonbinary identities, who are often excluded in similar analyses [16,45].
2) **Name-based approach.** We hypothesized our ability to find author pronouns would be diminished for authors outside the United States, given that sharing pronouns is not a global practice. Therefore, we used the *nomquamgender* python package [44] to assign a probability *p(gf)* that each author was gendered female. The package uses a “dictionary” of name-gender associations from more than 150 countries to assign a *p(gf)* value to an individual based on their name. A user can then set a threshold to classify binary gender based on *p(gf)*. We used the default threshold of 0.1, meaning we classified an author as *gendered female* if *p(gf)* was ≥ 0.90 and as *gendered male* if *p(gf)* was ≤ 0.10. If *p(gf)* was between 0.10 and 0.90, we classified an author as *uncertain*. Names that do not occur in the dictionary of name-gender associations cannot be assigned *p(gf)* values; we classified these names as *undetermined*.

We assessed concordance between gender classifications made using the pronouns-based approach versus the name-based approach and made a final gender classification list by merging the results of our two approaches, deferring to gender based on pronouns in cases of disagreement. When we were unable to determine gender by either approach, we classified author gender as *unknown*. We also attempted to collect voluntarily-disclosed demographic information about EHA employees from the organization’s human resources department to serve as a better validation dataset for our two-pronged approach to gender classification, but were unable to obtain this information before EHA closed in 2025. We acknowledge that all methods for classifying author gender are imperfect, and we may have incorrectly classified the gender of some authors. However, our two-pronged approach allowed us to maximize the data available for analysis and include genders beyond the binary. Because gender identity can be a sensitive topic, all gender-related data were stored only in our project database, with access restricted to project personnel. We deposited a de-identified database for public use on Zenodo [46].

### Analyses of gender, geography, income, and authorship

Analyses were performed in the R statistical environment v 4.4.2 [47]. We first calculated the percent of all FLAs across all combinations of author position and gender. We also calculated the number of FLAs associated with each unique author, and explored whether author productivity (here, assessed by the number of FLAs) differed by gender. For highly productive authors (those with ≥ 10 total FLAs), we calculated the percent of their FLAs composed of last authorships.

To examine interactions of geography and gender, we compared the number of authorships by gendered male and gendered female authors within each country. We calculated the percent of all FLAs across all combinations of author position and country income and all combinations of author position, gender, and country income.

We used a linear model to explore if author position, country income, and year were correlated with the annual percent of authorships by gendered female authors. Explanatory variables for the model included an interaction between year and author position, an interaction between country income and author position, and main effects of year, author position, and country income. Based on previous work exploring authorship trends over time in ecology and related fields [15,17,48], we expected that the percent of authorships by gendered female authors would increase over time, but the rate of increase would be lower for last authorships. Last authorships are often reserved for senior scientists (e.g., principal investigators), and promotion to this senior role typically takes years. Based on preliminary visual exploration of the data, we also expected the relationship between author position and the percent of authorships by gendered female authors to differ for countries of different income. Due to limited authorship data for low-income, lower-middle-income, and upper-middle-income countries in our dataset (see Results), we collapsed these three categories into one and treated country income as a binary variable in the model (high-income versus low- and middle-income). We treated year as continuous and author position as binary (first/last). We inspected model diagnostic plots to check that assumptions underlying linear regression were met (S1 Fig).

For research taking place outside of the United States (where EHA was headquartered), we explored the association between the geographic focus of an article and the geographic affiliations of the first and last authors. We did this to assess how often locally-based researchers received credit in the form of prestigious FLAs for their critical roles in these research projects. We first restricted the table of articles to exclude those with a “non-specific” article geography and those with an article geography that included the United States. We then calculated how often authorship geography “matched” article geography for the i) first author position only, ii) the last author position only, iii) either author position, and iv) both author positions. If multiple countries were listed for article geography (e.g., because fieldwork took place in several locations), we counted a match if the authorship geography was the same as any of those countries. To understand if authorship practices changed over time, we then repeated these calculations for two time periods: 2011–2016, and 2017–2022.

### Network analyses to examine gender and author centrality

Network analyses are commonly used to reveal structural aspects of social relationships, and centrality measures are designed to identify individuals within a network who are important to its structure [49]. In the context of co-authorship networks, these individuals tend to be senior researchers or highly-cited individuals [50]. To examine collaborations between authors, we calculated centrality measures for the network of all FLAs in the corpus. In this analysis, articles and authors represent two components of a bipartite graph, where authors are linked by co-authorship on an article. Because we were interested in connections between authors, we re-projected the bipartite graph such that it contained weighted edges between authors (nodes) based on their co-authorships. We then used the author network to explore the relationship between gender and two measures of centrality: *betweenness centrality* and *harmonic centrality*.

Betweenness centrality measures the number of shortest paths on which a node resides [51], where the shortest path is the walk between two nodes that requires traversing the least number of edges. A node with high betweenness centrality is structurally important to the graph. In our analysis, authors with high betweenness centrality likely represent people in positions of “power” within the network (e.g., principal investigators, those who control resources). Harmonic centrality measures the degree of a node (i.e., number of connections to other nodes) and its neighbors, up to a certain distance [52]. The higher the degree of a node and its neighbors, the higher the harmonic centrality–with the important caveat that the influence of neighbors decays with distance and is not inflated for unconnected subcomponents of the graph [49]. This provides information about how connected nodes and their neighbors are to the rest of the graph. In our analysis, an author with high harmonic centrality is collaborating with many people who are also collaborating with many people.

We calculated betweenness centrality and harmonic centrality for each author using the *igraph* R package [53,54]. We also calculated mean betweenness centrality and mean harmonic centrality for each gender group, as well as 95% high density confidence intervals (HDCIs) for each measure. We calculated 95th percentile values for betweenness centrality and harmonic centrality and used these as cutoffs to tally the number of highly powerful and highly collaborative authors according to gender. Finally, we created two depictions of the authorship network using node color to represent author gender and node size to represent each centrality measure.

## Results

### Dataset summary

Filtering the EHA research outputs catalog to peer-reviewed journal articles published from January 1, 2011 to December 31, 2022 resulted in a corpus of 451 articles. Articles in the corpus were most commonly published in PLOS ONE (*n* = 40) and EcoHealth (*n* = 38), followed by Viruses (*n* = 11), mBio (*n* = 9), PNAS (*n* = 8), and Transboundary and Emerging Diseases (*n* = 8). We identified 898 FLAs associated with those 451 articles, which were linked to 498 unique authors, 249 institutions, and 43 countries.

### Gender composition of authors and authorships

After combining the results of our two gender classification approaches (see S2 Text and S1 Table for a comparison of the approaches), prioritizing gender inferred using pronouns in cases of dataset disagreement, we ultimately classified 297 authors (59.6%) as gendered male, 186 (37.3%) as gendered female, 1 (0.2%) as gendered nonbinary, and 14 (2.8%) as unknown gender. Of all FLAs, 584 (65.0%) were by gendered male authors, 295 (32.9%) were by gendered female authors, 1 (0.1%) was by a gendered nonbinary author, and 18 (2.0%) were by authors of unknown gender. Our gender classification process was least successful for authorships listing an affiliation with China, with 16 out of 44 authorships for this country classified as unknown gender. Given that only one author was classified as gendered nonbinary, we included that author in figures (except where noted) but did not attempt to draw any conclusions about authorship patterns in regard to gendered nonbinary authors.

**Fig 1:**
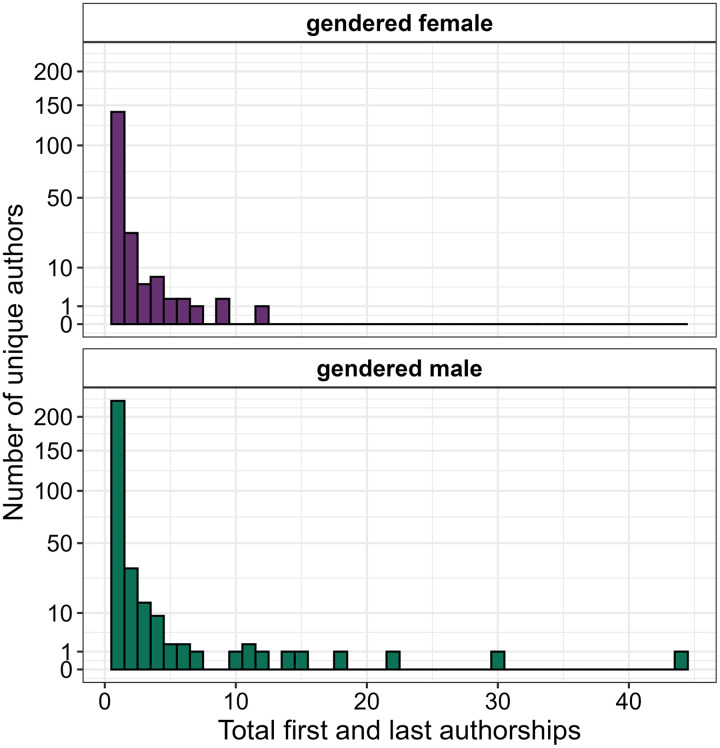
Total first and last authorships associated with unique authors. Gendered female (*n* = 186) and gendered male (*n* = 297) authors are displayed in the top and bottom panels respectively. Authors classified as gendered nonbinary (*n* = 1) or unknown gender (*n* = 14) are not displayed. Note that the y-axis is on a square-root scale.

For both the first and last author positions, there were more authorships by gendered male authors than by gendered female authors (first-male: 29.3% of all FLAs, first-female: 19.5%, last-male: 35.7%, last-female: 13.4%; S2 Fig). Nearly three-quarters of all authors (73.7%) had just one authorship each (226 gendered male authors and 141 gendered female authors) (Fig 1). There was only one gendered female author with ≥ 10 total FLAs, while there were ten gendered male authors with 10–44 total FLAs each. Collectively, those ten gendered male authors accounted for 187 (20.8%) of all FLAs in the corpus (Fig 1). Last authorships made up >65% of FLAs for eight of the eleven most productive authors (S2 Table).

### Intersections of gender, country income, and author position

A total of 43 countries (high-income: *n* = 19, upper-middle-income: *n* = 8, lower-middle-income: *n* = 13, low-income: *n* = 3) were represented in authorship affiliations (S3 Fig). Most authorships listed an affiliation with the United States (49.1%), Bangladesh (12.1%), Australia (9.2%), China (4.9%), or the United Kingdom (4.2%), together comprising 79.5% of all FLAs. Within each of these five countries, there were more authorships by gendered male authors than authors of any other gender (S4 Fig). Most FLAs had an HIC affiliation (71.5%), while other income groups were less well represented (upper-middle: 12.0%, lower-middle: 15.1%, low: 1.3%; S5 Fig).

**Fig 2:**
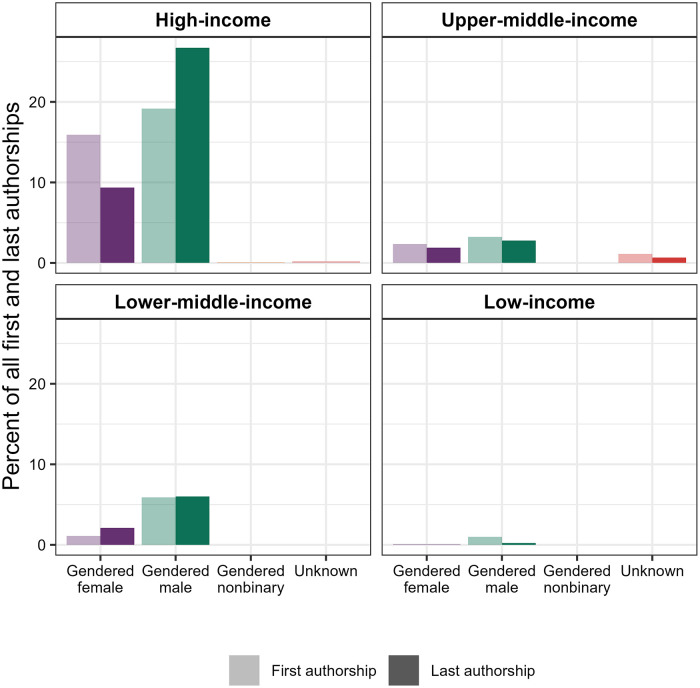
Percent of all first and last authorships (*n* = 898) separated by country income. Colors indicate author gender and shading indicates author position. A total of 43 countries were represented in first and last authorship affiliations (high-income: *n* = 19, upper-middle-income: *n* = 8, lower-middle-income: *n* = 13, low-income: *n* = 3).

For authorships with an HIC affiliation, there was an interplay between gender and author position (Fig 2). Specifically, there were more first authorships than last authorships (15.9% versus 9.4% of all FLAs) by gendered female authors, while there were more last than first authorships (26.7% versus 19.2% of all FLAs) by gendered male authors. The data on authorships with an upper-middle-income, lower-middle-income, or low-income country affiliation were too limited to determine if differences by gender and author position were meaningful (Fig 2).

### Gender composition of authorships over time

**Fig 3:**
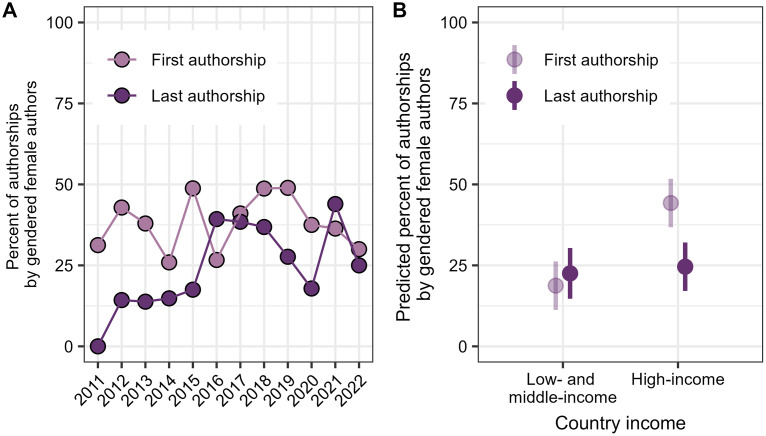
Observed and model-predicted gendered female authorship. **(A)** Observed percent of authorships by gendered female authors over time, separated by author position. First authorships by gendered female authors/Total first authorships (2011–2022): 5/16, 9/21, 11/29, 7/27, 20/41, 8/30, 16/39, 19/39, 23/47, 21/56, 24/66, 12/40. Last authorships by gendered female authors/Total last authorships (2011–2022): 0/16, 3/21, 4/29, 4/27, 7/40, 11/28, 15/39, 14/38, 13/47, 10/56, 29/66, 10/40. **(B)** Model-predicted percent of authorships by gendered female authors, separated by author position and country income.

The percent of first authorships by gendered female authors fluctuated from year to year, displaying no clear trend over time (Fig 3A). The percent of first authorships by gendered female authors never dipped below 25% nor reached above 50% over the 12-year timespan of our dataset. In contrast, there appeared to be a positive trend over time for last authorships by gendered female authors (Fig 3A). In 2011, there were zero last authorships by gendered female authors. This value jumped to 14.3% in 2012, remained fairly constant through 2015, and jumped again to 39.3% in 2016. There was a subsequent monotonic decline to 17.9% by 2020. The percent of last authorships jumped to a maximum of 43.9% in 2021, then decreased to 25.0% in 2022.

A linear model explaining the percent of authorships by gendered female authors found that the interaction between year and author position was not statistically significant (*β* = 1.45, *SE* = 1.11, *P* = 0.20; S3 Table). The main effect of year was positive, indicating an overall increase in authorships by gendered female authors, though this was also not statistically significant (*β* = 0.85, *SE* = 0.76, *P* = 0.27). There was a significant interaction between author position and country income (*β* = −23.45, *SE* = 7.49, *P* = 0.0032), meaning the relationship between author position (first or last) and the percent of authorships by gendered female authors in an author position depended on country income. For HICs, predicted authorships by gendered female authors were greater for first authorships (44.3%, 95% confidence interval: 36.8–51.7) compared to last authorships (24.6%, 95% CI: 17.1–32.1; Fig 3B). However, for LMICs, the predicted first (18.74%, 95% CI: 11.3–26.2) and last (22.5%, 95% CI: 14.7–30.4) authorships by gendered female authors were similar. Accounting for the number of variables, the model explained 41% of the variance in the outcome variable.

### Alignment of authorship geography and article geography

We found that 280 articles (62.1% of all articles in the corpus) were geographically focused on one or more non-US countries (e.g., where field or laboratory work was performed or specialized knowledge of a country was required). About two-thirds of the time (63.9%, 179/280), either the first or last authorship geography matched the article geography (S4 Table). First authorship geography matched article geography 53.6% of the time, whereas last authorship geography matched article geography 47.1% of the time. Both first and last authorships matched the article geography 36.7% of the time. When first authorship geography did not match article geography, the first authors listed a United States or other HIC affiliation 96% of the time; upper-middle-income country affiliations were rarely listed (4%). Similarly, when last authorship geography did not match article geography, the last author listed a United States or other HIC affiliation 97% of the time; upper-middle-income (1%) or lower-middle-income (2%) country affiliations were rarely listed. Though there were more articles published in the second half of the dataset (2011–2016: 95 articles; 2017–2022: 185 articles), the rates of authorship-geography matches stayed nearly constant over time (S4 Table). There were 142 articles (31.5%) with a non-specific article geography and 29 articles (6.4%) with an article geography that included the United States.

### Characteristics of the author network

**Fig 4:**
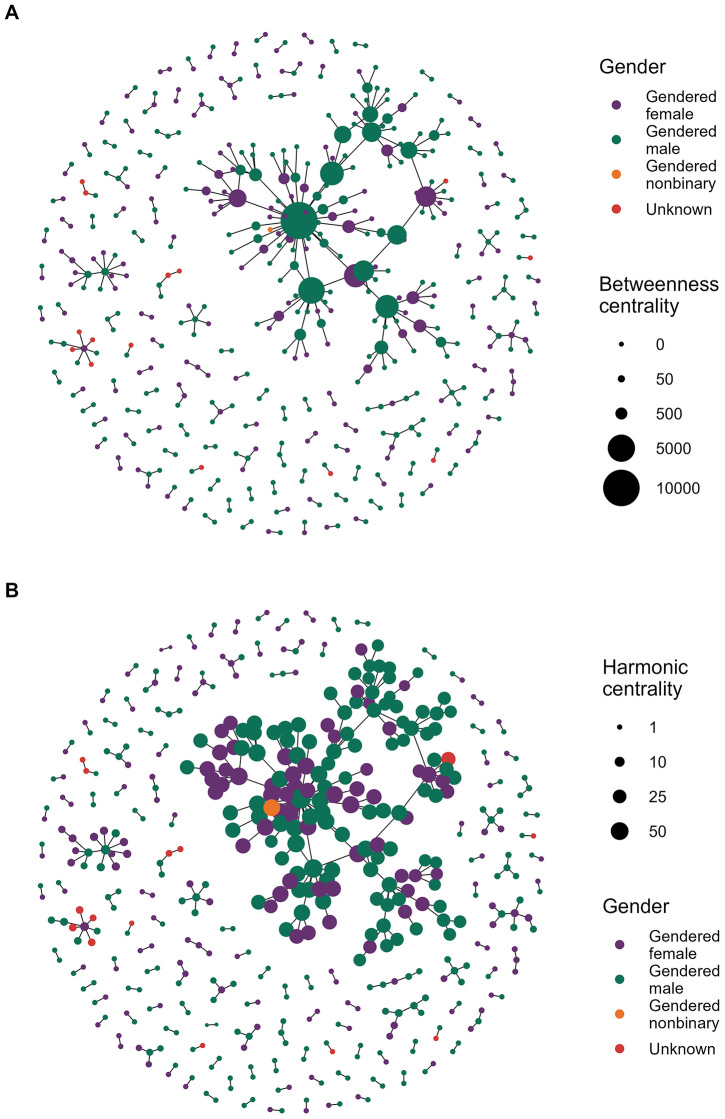
Depictions of the author network by two measures of centrality. In both panels, node (author) color indicates gender. **(A)** Network with node size representing betweenness centrality, which conveys information about the structural importance of an individual to the overall network. **(B)** Network with node size representing harmonic centrality, which conveys information about how connected an individual is to the rest of the graph.

We created two author network depictions to show relationships between author gender and our two centrality measures of interest (Fig 4). The mean betweenness centrality of the gendered male author group was almost double that of the gendered female author group (129.21 versus 64.84; S5 Table), indicating that on average, gendered male authors were more structurally important to the network. There were nearly twice as many gendered male authors in positions of “power” in the network (betweenness centrality ≥ 317, the 95th percentile) compared to gendered female authors (17 versus 9). In contrast, the two author groups had similar mean harmonic centrality (gendered male: 11.51, gendered female: 11.25; S5 Table), indicating similar levels of connection or collaboration. There were 12 gendered female authors, 15 gendered male authors, and one gendered nonbinary author who were highly collaborative (harmonic centrality ≥ 43.1, the 95th percentile).

## Discussion

We analyzed authorship patterns within the extended publication network of EcoHealth Alliance, a United States-based organization that conducted One Health and conservation research. Within our corpus of 451 peer-reviewed journal articles, we found that gendered male authors were dominant in multiple aspects: they represented ~60% of all authors, 65% of all FLAs, and 91% of highly productive authors. The last author position was particularly male-dominated, with 2.66 times as many last authorships by gendered male authors as by gendered female authors. Gendered male authors were more structurally important to the author network on average and comprised 65% of highly “powerful” authors in the network. HICs were also overrepresented, with ~72% of FLAs listing an HIC affiliation.

We focused our analyses on first and last authorships because these are typically perceived as prestigious in STEM fields [9,10]. In the ecological and environmental sciences (major constituent fields within One Health), the first author is commonly viewed as taking the lead role in study conceptualization, data collection and analysis, and manuscript writing, whereas the last author is often viewed as the “senior” author whose work or role made the study possible [55–58]. Our finding of more first than last authorships for gendered female authors (a pattern which has been previously observed in ecology and conservation; [16,48,59]), but the reverse pattern for gendered male authors, suggests gendered female authors were more likely to lead papers whereas gendered male authors were more likely to lead research groups. This is supported by our finding that the most productive authors in our dataset (i.e., those with ≥ 10 total FLAs) were nearly all gendered male (10/11), with last authorships comprising a large percentage of their FLAs. Leading a publication as a first author is a time-consuming endeavor requiring intensive analysis and writing; therefore, the overrepresentation of gendered female authors in this role may result in decreased overall productivity. In contrast, research supervisors or principal investigators can accumulate last authorships on their team members’ publications for comparatively less effort per publication (e.g., general oversight and manuscript editing). It is important to note that the overall authorship pattern we observed (more first than last authorships for gendered female authors, but more last than first authorships for gendered male authors) was driven primarily by authorships with HIC affiliations.

The interaction between country income and author position was also an important explanatory variable in our linear model explaining the percent of authorships by gendered female authors. For HICs, there was a striking disconnect in the model-predicted percent of first authorships (~44%) versus last authorships (~25%) by gendered female authors (Fig 3B). This could represent a lack of career advancement for gendered female authors, who may publish primarily first authorships as early or mid-career researchers but rarely become senior researchers with a shift to publishing primarily last authorships.

Our network analysis showed gendered male authors were disproportionately represented in structurally important positions based on betweenness centrality scores. However, gendered male and gendered female authors were similarly collaborative: there were similar numbers of gendered male and gendered female authors in the top 5th percentile of harmonic centrality scores, and average harmonic centrality scores for the two gender groups were nearly identical. Together, these results reveal gendered female authors were just as collaborative as their gendered male peers, but it was gendered male authors who were more likely to be in positions of power (i.e., those who control or distribute resources like funding).

Though EHA primarily conducted research in LMICs and aimed to engage local partners, credit in the form of prestigious first and last authorships went to authors affiliated with the United States or another HIC over half of the time. When we examined articles that were geographically focused outside the United States—representing an opportunity for local leadership—first and last authorship geography each matched the article geography about half the time. In ~36% of articles, neither the first nor the last authorship geography matched the article geography. These findings echo previous studies showing when research takes place in or involves participants from an LMIC, researchers affiliated with that country are only included as first or last authors about half the time [24–26]. One reason for this disparity could be the devaluation of certain steps in the scientific process (e.g., project implementation, data collection) that are usually conducted by LMIC researchers in comparison to others typically conducted by HIC researchers (e.g., drafting a manuscript, acquiring research funding) [60]. Concerns about the potential for editorial bias, where journals may favor well-known authors or those from English-speaking countries, might also lead LMIC researchers to cede first or last authorship to HIC collaborators to increase the chance an article will be accepted for publication [60]. Authors from HICs may prioritize first or last authorships if they believe this will improve their chances of securing future research funding [6].

## Conclusion

Within our corpus, we found that gendered male researchers and researchers with HIC affiliations were often in prestigious author positions. Though our analysis was based on articles with at least one EHA-affiliated author, authorship affiliations in the corpus extended to nearly 250 institutions across 43 countries, demonstrating the broad scope of EHA’s partnerships. Our findings align with a case study of The Nature Conservancy, which found that men represented 58% of author and 68% of authorships on TNC publications, and that nearly 90% of authors were located in the Global North [16]. Similarly, a study of authorship in the field of infectious disease dynamics showed that men were overrepresented as first and last authors compared to the global STEM workforce and global population, and that most last authors were located in the Global North [61]. Neither our study, nor the two mentioned above, used a research methodology that could determine how and why author order was assigned (e.g., interviews, surveys, review of documents that describe authorship determination); future work of this nature would be valuable. However, we offer recommendations—informed by the patterns observed in our data and based on our personal experiences as researchers—that we believe would help address the gender and geography disparities in authorship we observed.

Scientists at organizations in HICs should not impose their own authorship norms when collaborating with peers from LMICs and should familiarize themselves with the norms of their collaborators. We recommend having early and ongoing conversations about authorship practices and expectations with the entire research team and capturing the outcome of those conversations as part of a formal document (e.g., a data management plan). Data collection practices can assist in measuring researchers’ contributions by documenting who conducted particular tasks. Following project completion, adopting a consensus-based decision making process and considering the different types of labor involved in a project, who performed that labor, and the “social location” of each author may be useful strategies for determining authorship status and order [62]. Attribution schemes like the CRediT taxonomy [27] can also help assess author contributions to a manuscript and are increasingly required by journals. Scientists should consider consulting academic support staff like research librarians who may have a broader perspective on author ordering practices and strategies for equitable acknowledgement.

We recommend organizations monitor the career progression and attrition of their researchers, particularly those with marginalized identities. Further, we encourage organizations to critically evaluate how leadership structure (including who is allowed or encouraged to be a principal investigator) can impact scientists’ publication records. To avoid powerful individuals gaining undue benefit from their structural position (e.g., principal investigators automatically having last authorship on papers generated within their lab groups), organizations could develop guidelines around author order recognizing the different types of contributions scientists make [63]. Furthermore, organizations should ensure proper resources are dedicated to communicating, accessing, and applying these tools, including providing training via research support staff, supplying institutional tools that integrate the guidelines like DMPTool (https://dmptool.org), and making the guidelines easily discoverable via web search. For HIC-LMIC collaborations, these organization-level authorship guidelines should explicitly encourage inclusion of authors from LMICs in first and last author positions [64]. Organizations in HICs may need to re-envision performance evaluation and promotion as a more holistic process that values research quality and also considers criteria such as the demonstrable use of equitable collaboration practices [65].

Finally, as gatekeepers at different points in the project life cycle, funders and publishers ultimately control what research is conducted, how it is conducted, and how findings are distributed. As such, they should take an active role in requiring researchers to reflect on and acknowledge contributions from all collaborators [66]. For example, the journal *BMJ Global Health* requires authors to provide a structured reflexivity statement [64] when submitting manuscripts involving HIC-LMIC collaborations. In 2021, PLOS implemented an Inclusivity in Global Research policy across all its journals asking researchers to, when relevant, complete a questionnaire that “outlines ethical, cultural, and scientific considerations specific to inclusivity in global research” (https://theplosblog.plos.org/2021/09/announcing-a-new-plos-policy-on-inclusion-in-global-research/). With the proliferation of digital object identifiers (DOIs), it is easier than ever for publishers to connect journal articles to other research artifacts. As such, publishers could require authors to include a DOI that links an article to a data management plan or other research artifact describing how authorship order was assigned. This would allow journal editors and peer-reviewers to compare alignment between proposed and actual authorship order. When evaluating a project proposal, funders should require equitable allocation of intellectual property and scholarly recognition between HIC and LMIC researchers. Funders should also account for structural biases that may have shaped applicants’ publication records when evaluating researchers’ capacity to conduct a project. Taken together, these actions by funders and publishers would create top-down pressure on researchers and organizations to improve equity in how research is conducted and published.

Though EcoHealth Alliance has closed since the initiation of this project, we hope our results will inspire changes in international research collaborations and authorship practices at similar organizations. Studies like ours can reveal previously unrecognized authorship patterns within an organization. By providing the code and detailed methodology to produce our findings, we offer a reproducible template for other organizations to assess their own authorship patterns. If more organizations conduct similar case studies, it could enable the research community to draw broader conclusions about authorship dynamics across domains.

## Supporting information

S1 Fig Model diagnostic plots. (DOCX)

S2 Fig Percent of all first and last authorships (*n* = 898) separated by author gender and author position. (DOCX)

S3 Fig Number of first and last authorships (*n* = 898) separated by country affiliation. Points are colored according to country income. Note that the x-axis is on a log-10 scale.
(DOCX)

S4 Fig Number of first and last authorships (*n* = 898) by country affiliation and gender. Data for the five countries with the most first and last authorships are displayed individually, while data for the remaining countries (*n* = 37) are grouped into “Other”.
(DOCX)

S5 Fig Percent of all first and last authorships (*n* = 898) separated by country income and author position. (DOCX)

S1 Table Comparison of a pronouns-based approach and a name-based approach to classify author genders. See *Gender classification of authors* in the main text for details of how authors were classified using each approach.
(DOCX)

S2 Table Last authorships as a percentage of all first and last authorships (FLAs) for the most productive authors in the dataset (i.e., ≥ 10 FLAs). (DOCX)

S3 Table Model coefficients for a linear model to examine effects of author position, country income, and year on the percent of authorships by gendered female authors. The “Year” variable was centered around 2011 to improve coefficient interpretability. *P* values < 0.05 are bolded.
(DOCX)

S4 Table Matches between authorship geography (i.e., country affiliation) and article geography (i.e., the geographic focus of a study, excluding the United States). A “match” occurred when the authorship geography was the same as any of the countries contained in the article geography. Values are provided for the whole timespan of the data (2011–2022) as well as broken down into two time periods (2011–2016 and 2017–2022) to explore potential changes in authorship over time. Denominator sizes are sometimes different because not all articles had a last authorship (i.e., sole-authored articles, which were counted as first authorships).
(DOCX)

S5 Table A summary of two measures of network centrality (betweenness centrality and harmonic centrality) calculated for authors separated by gender. HDCI = high density confidence interval.
(DOCX)

S1 Text Creation of the corpus. (DOCX)

S2 Text Comparison of two approaches to classify author gender. (DOCX)
