# Supplementary figures and images for: Gendered male and high-income country authors dominate publication at a One Health research organization
Source: PLoS One. 2026 Jun 26;21(6):e0352401. doi: 10.1371/journal.pone.0352401 (PMC13308861; doi:10.1371/journal.pone.0352401)

**Fig. S1. Model diagnostic plots.**


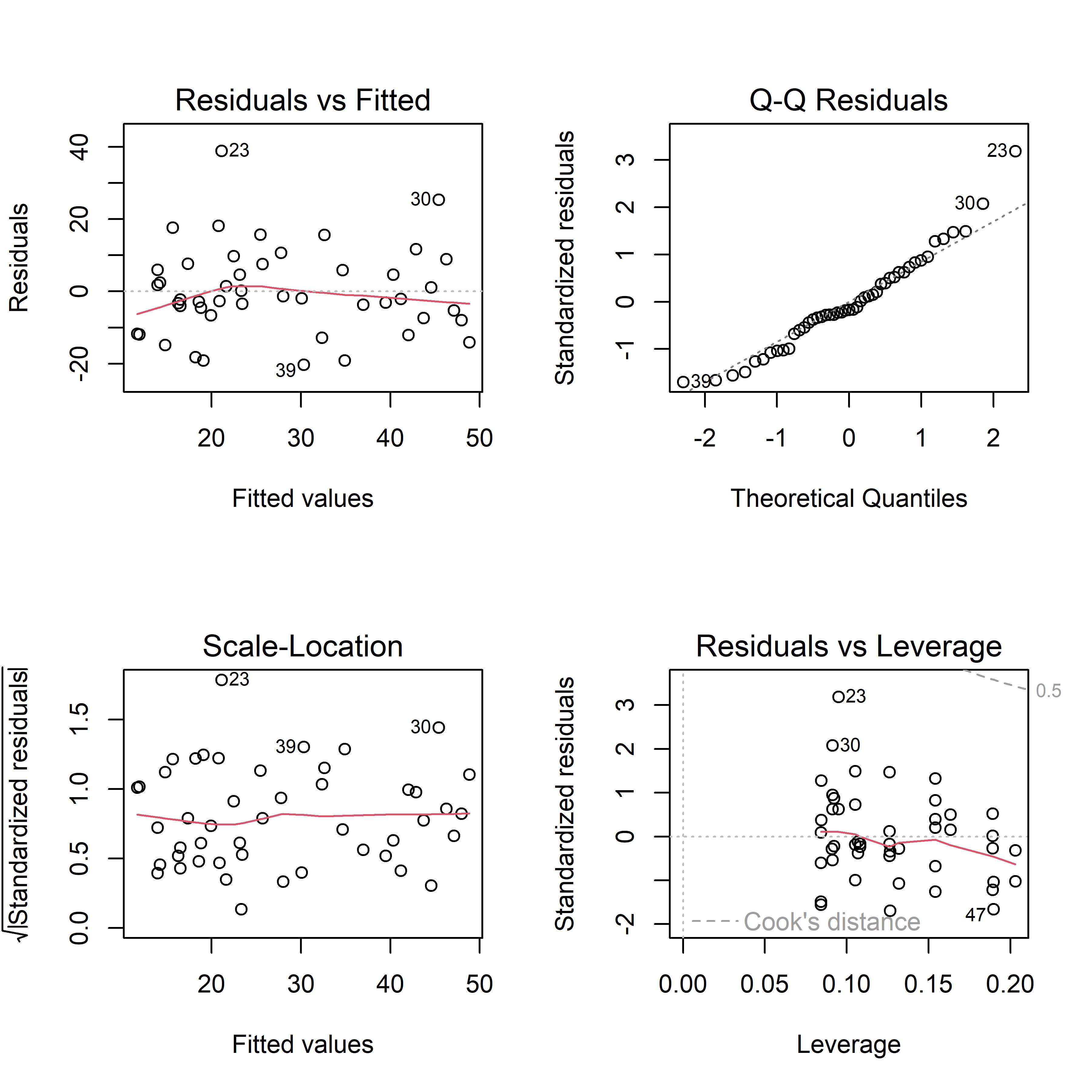

Supplement: S1 Fig — (DOCX) [file pone.0352401.s001.docx]
