# Supplementary material for: Gendered male and high-income country authors dominate publication at a One Health research organization
Source: PLoS One. 2026 Jun 26;21(6):e0352401. doi: 10.1371/journal.pone.0352401 (PMC13308861; doi:10.1371/journal.pone.0352401)

**Fig. S3. Number of first and last authorships (*n* = 898) separated by country affiliation.** Points are colored according to country income. Note that the x-axis is on a log-10 scale.


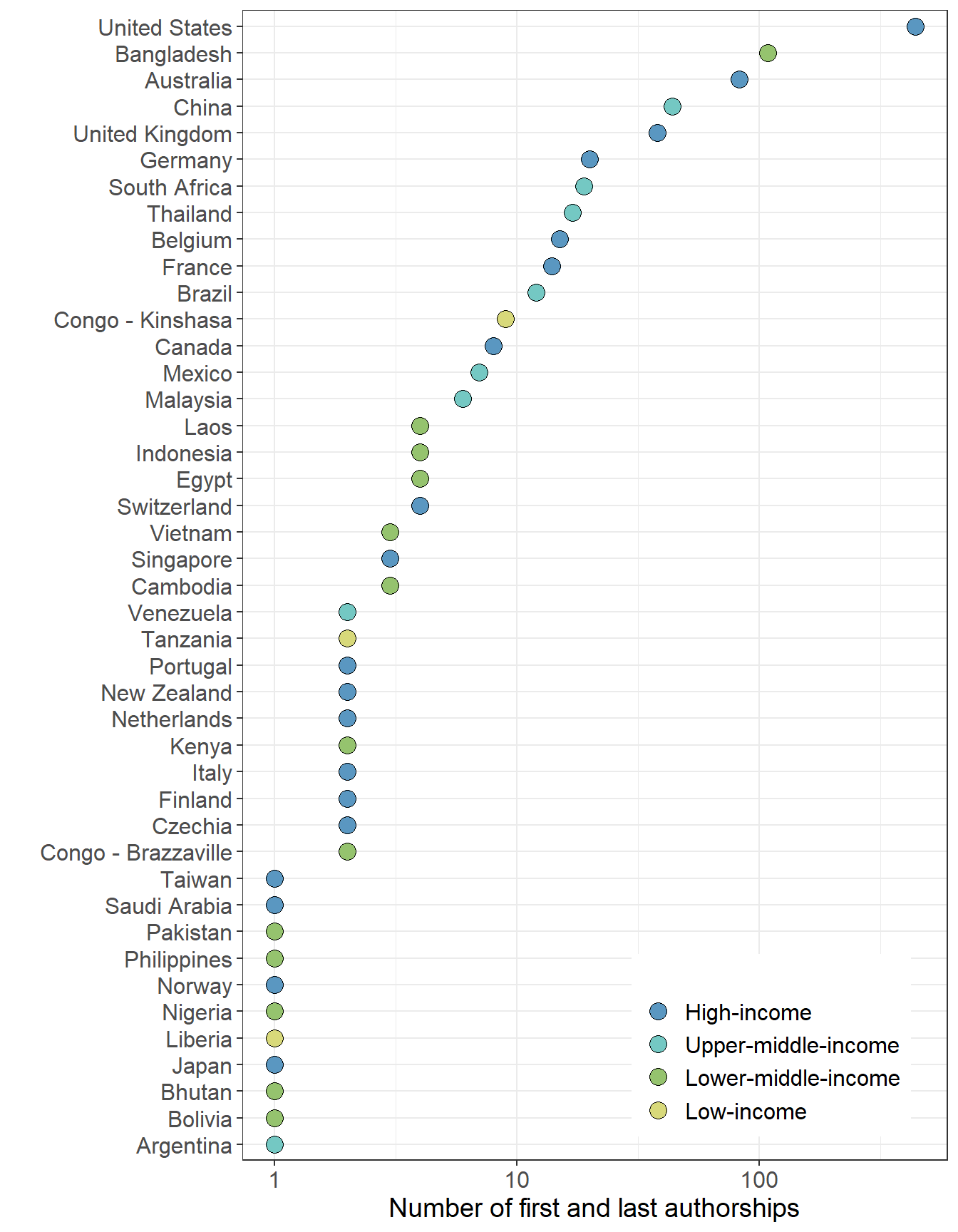

Supplement: S3 Fig — Points are colored according to country income. Note that the x-axis is on a log-10 scale. (DOCX) [file pone.0352401.s003.docx]
