# Supplementary material for: Gendered male and high-income country authors dominate publication at a One Health research organization
Source: PLoS One. 2026 Jun 26;21(6):e0352401. doi: 10.1371/journal.pone.0352401 (PMC13308861; doi:10.1371/journal.pone.0352401)
Supplement: S4 Fig — Data for the five countries with the most first and last authorships are displayed individually, while data for the remaining countries (n = 37) are grouped into “Other”. (DOCX) [file pone.0352401.s004.docx]

**Table S4. Matches between authorship geography (i.e. country affiliation) and article geography (i.e. the geographic focus of a study, excluding the United States).** A “match” occurred when the authorship geography was the same as any of the countries contained in the article geography. Values are provided for the whole timespan of the data (2011-2022) as well as broken down into two time periods (2011-2016 and 2017-2022) to explore potential changes in authorship over time. Denominator sizes are sometimes different because not all articles had a last authorship (i.e. sole-authored articles, which were counted as first authorships).

| **Time period** | **First authorship match** | **Last authorship match** | **Either match** | **Both match** |
| --- | --- | --- | --- | --- |
| 2011-2022 | 150/280 (53.6%) | 131/278 (47.1%) | 179/280 (63.9%) | 102/278 (36.7%) |
| 2011-2016 | 51/95 (53.7%) | 43/94 (45.7%) | 61/95 (64.2%) | 33/94 (35.1%) |
| 2017-2022 | 99/185 (53.5%) | 88/184 (47.8%) | 118/185 (63.8%) | 69/184 (37.5%) |
