# Supplementary material for: Gendered male and high-income country authors dominate publication at a One Health research organization
Source: PLoS One. 2026 Jun 26;21(6):e0352401. doi: 10.1371/journal.pone.0352401 (PMC13308861; doi:10.1371/journal.pone.0352401)

**Fig. S5. Percent of all first and last authorships (*n* = 898) separated by country income and author position.**


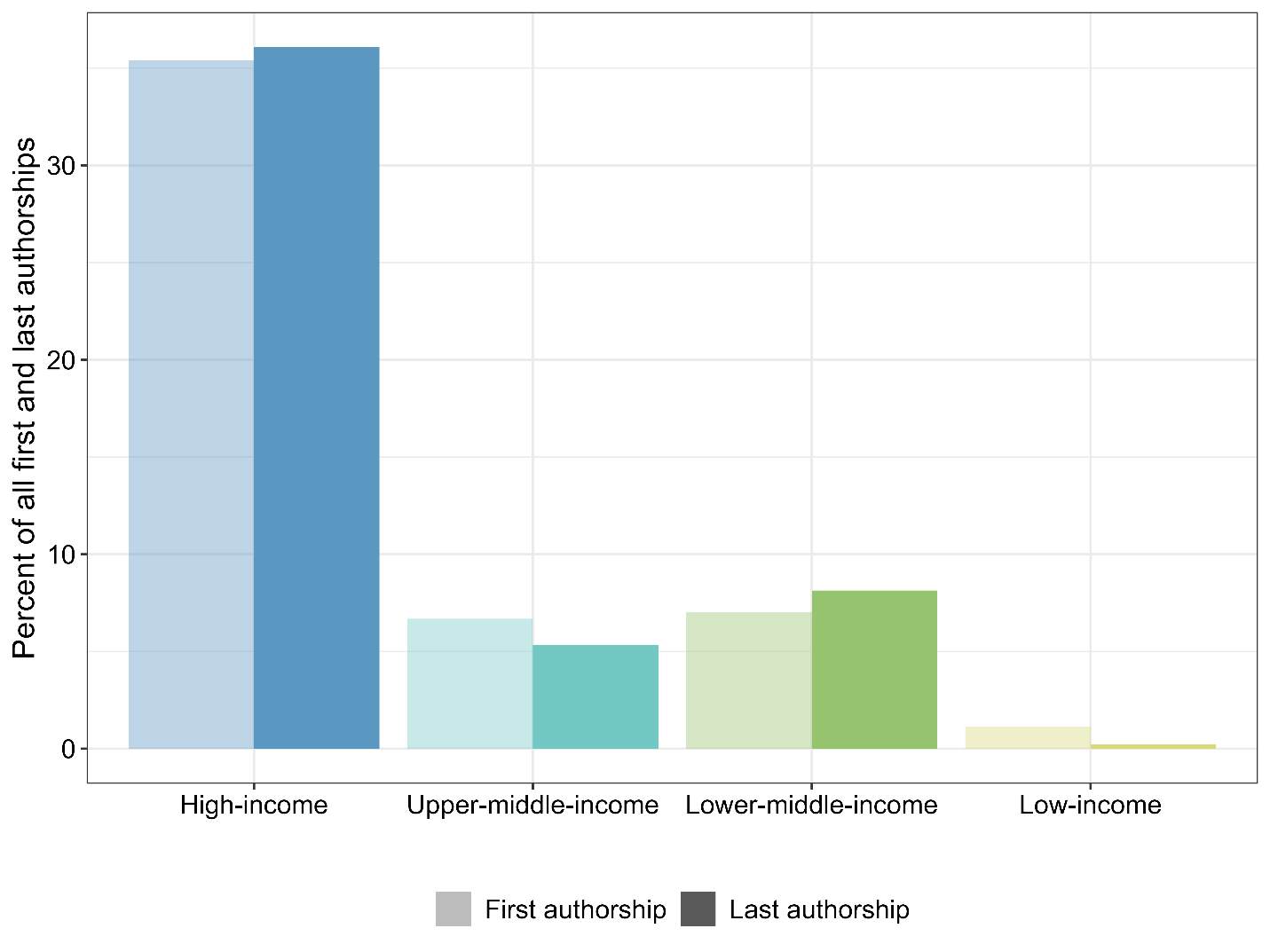

Supplement: S5 Fig — (DOCX) [file pone.0352401.s005.docx]
