# Supplementary material for: Gendered male and high-income country authors dominate publication at a One Health research organization
Source: PLoS One. 2026 Jun 26;21(6):e0352401. doi: 10.1371/journal.pone.0352401 (PMC13308861; doi:10.1371/journal.pone.0352401)
Supplement: S1 Table — See Gender classification of authors in the main text for details of how authors were classified using each approach. (DOCX) [file pone.0352401.s006.docx]

**Table S1.** **Comparison of a pronouns-based approach and a name-based approach to classify author genders.** See *Gender classification of authors* in the main text for details of how authors were classified using each approach.

|  | | Gender classification based on name | | | |
| --- | --- | --- | --- | --- | --- |
|  |  | *gendered female* | *gendered male* | *uncertain* | *undetermined* |
| Gender classification based on pronouns | *gendered female* | 151 | 3 | 20 | 7 |
|  | *gendered male* | 3 | 244 | 24 | 9 |
|  | *gendered nonbinary* | 0 | 1 | 0 | 0 |
|  | *undetermined* | 5 | 17 | 12 | 2 |
