# Supplementary material for: Gendered male and high-income country authors dominate publication at a One Health research organization
Source: PLoS One. 2026 Jun 26;21(6):e0352401. doi: 10.1371/journal.pone.0352401 (PMC13308861; doi:10.1371/journal.pone.0352401)
Supplement: S2 Table — (DOCX) [file pone.0352401.s007.docx]

**Table S2. Last authorships as a percentage of all first and last authorships (FLAs) for the most productive authors in the dataset (i.e. ≥ 10 FLAs).**

| **Total FLAs** | **Author gender** | **Last authorships/total FLAs (%)** |
| --- | --- | --- |
| 44 | Gendered male | 88.6 |
| 30 | Gendered male | 43.3 |
| 22 | Gendered male | 68.2 |
| 18 | Gendered male | 77.8 |
| 15 | Gendered male | 53.3 |
| 14 | Gendered male | 92.9 |
| 12 | Gendered female | 100 |
| 12 | Gendered male | 100 |
| 11 | Gendered male | 18.2 |
| 11 | Gendered male | 72.7 |
| 10 | Gendered male | 70 |
