# Supplementary material for: Gendered male and high-income country authors dominate publication at a One Health research organization
Source: PLoS One. 2026 Jun 26;21(6):e0352401. doi: 10.1371/journal.pone.0352401 (PMC13308861; doi:10.1371/journal.pone.0352401)
Supplement: S3 Table — The “Year” variable was centered around 2011 to improve coefficient interpretability. P values < 0.05 are bolded. (DOCX) [file pone.0352401.s008.docx]

**Table S3. Model coefficients for a linear model to examine effects of author position, country income, and year on the percent of authorships by gendered female authors.** The “Year” variable was centered around 2011 to improve coefficient interpretability. *P* values < 0.05 are bolded.

| **Variable** | **Estimate** | **SE** | **t** | ***P*** |
| --- | --- | --- | --- | --- |
| Intercept | 13.97 | 5.57 | 2.51 | **0.016** |
| Year | 0.85 | 0.76 | 1.12 | 0.27 |
| Author position(Last) | -4.35 | 8.33 | -0.52 | 0.60 |
| Country income(High) | 25.51 | 5.23 | 4.87 | **1.7e-5** |
| Year : Author position(Last) | 1.45 | 1.11 | 1.31 | 0.20 |
| Author position(Last) : Country income(High) | -23.45 | 7.49 | -3.13 | **0.0032** |
