# Supplementary material for: Gendered male and high-income country authors dominate publication at a One Health research organization
Source: PLoS One. 2026 Jun 26;21(6):e0352401. doi: 10.1371/journal.pone.0352401 (PMC13308861; doi:10.1371/journal.pone.0352401)
Supplement: S5 Table — HDCI = high density confidence interval. (DOCX) [file pone.0352401.s010.docx]

**Table S5. A summary of two measures of network centrality (betweenness centrality and harmonic centrality) calculated for authors separated by gender.** HDCI = high density confidence interval.

| **Gender** | **Group size** | **Mean betweenness centrality** | **Betweenness centrality 95% HDCI** | **Mean harmonic centrality** | **Harmonic centrality 95% HDCI** |
| --- | --- | --- | --- | --- | --- |
| Gendered female | 186 | 64.84 | 0-190.6 | 11.25 | 0.65-43.12 |
| Gendered male | 297 | 129.21 | 0-317.0 | 11.51 | 0.83-43.12 |
| Gendered nonbinary | 1 | 0 | – | 43.12 | -- |
| Unknown | 14 | 0.21 | 0-2.0 | 3.95 | 1.00-28.51 |
