# Supplementary material for: Gendered male and high-income country authors dominate publication at a One Health research organization
Source: PLoS One. 2026 Jun 26;21(6):e0352401. doi: 10.1371/journal.pone.0352401 (PMC13308861; doi:10.1371/journal.pone.0352401)
Supplement: S1 Text — (DOCX) [file pone.0352401.s011.docx]

**Text S1**

**Creation of the corpus**

Prior to initiating this study, EHA developed a catalog of research outputs associated with the organization for use in tracking internal metrics. Research outputs consisted mainly of scientific journal articles, but also included materials such as conference abstracts, book chapters, datasets, reports, and preprints. Research outputs and associated authorship records were imported into this catalog via an OpenAlex API query using the *openalex* R package [1, 2], when the authorship institution attribute for at least one author contained the Research Organization Registry (<https://ror.org/>) identifier for EHA. Records were processed digitally to keep pertinent fields, identify potential duplicates, and store them in an Airtable (<https://airtable.com/>) database. Each research output was associated with metadata including title, publication date, author names, and author affiliations (i.e. organization and country).

For this study, we exported a copy of EHA’s research outputs catalog to a separate Airtable database, accessible only to project personnel. We filtered the dataset to peer-reviewed journal articles published from January 1, 2011 to December 31, 2022. We chose the start date to align with the shift in the organization’s name and research focus. We note that “gray” and “white” literature (e.g. graduate theses, government reports, policy documents, technical reports) also represent important research outputs and require substantial time and effort to produce. However, we focused on peer-reviewed literature following past work [3] and because gray and white literature may have different authorship norms. Records were manually reviewed and cleaned to ensure accuracy. Henceforth, we refer to this set of peer-reviewed, EHA-affiliated journal articles as the *corpus*.

**References**

1. Priem J, Piwowar H, Orr R. OpenAlex: A fully-open index of scholarly works, authors, venues, institutions, and concepts. arXiv. 2022:2205.01833.

2. Aria M, Le T, Cuccurullo C, Belfiore A, Choe J. openalexR: An R-Tool for Collecting Bibliometric Data from OpenAlex. The R Journal. 2024;15(4):167-80.

3. James R, Ariunbaatar J, Bresnahan M, Carlos-Grotjahn C, Fisher JRB, Gibbs B, et al. Gender and conservation science: Men continue to out-publish women at the world's largest environmental conservation non-profit organization. Conservation Science and Practice. 2022;4(8):e12748.
