# Supplementary material for: Gendered male and high-income country authors dominate publication at a One Health research organization
Source: PLoS One. 2026 Jun 26;21(6):e0352401. doi: 10.1371/journal.pone.0352401 (PMC13308861; doi:10.1371/journal.pone.0352401)
Supplement: S2 Text — (DOCX) [file pone.0352401.s012.docx]

**Text S2**

**Comparison of two approaches to classify author gender**

Using the pronouns-based approach, we classified 280 authors (56.2%) as gendered male, 181 (36.3%) as gendered female, and 1 (0.2%) as gendered nonbinary. We were unable to find pronouns for 36 authors (7.2%). Of the 462 authors whose pronouns were identified, publicly available online information was the source for 323 (69.9%), while professional interactions were the source for the remaining 139 (30.1%). Using the name-based approach, we classified 265 authors (53.2%) as gendered male, 159 (31.9%) as gendered female, 56 (11.2%) as uncertain, and 18 (3.6%) as undetermined. Agreement between the two gender classification approaches was generally high (Table S1); out of 498 authors, both approaches classified 151 authors as gendered female and 244 authors as gendered male. There were four cases where an author was gendered female or gendered nonbinary based on pronouns but gendered male based on name, and three cases where an author was gendered male based on pronouns but gendered female based on name.
